# Tex15 is required for vomeronasal sensory neuron diversity and male pheromone detection

**DOI:** 10.64898/2025.12.03.690614

**Published:** 2025-12-10

**Authors:** Nader Boutros Ghali, Paige Kramer, Ryan Edwards, Irina Ruzina, Hetanshi Patel, Nusrath Yusuf, Zain Zaidi, Kevin Monahan

**Affiliations:** 1 Department of Molecular Biology and Biochemistry, Rutgers University, Piscataway, NJ, 08904, USA

## Abstract

The mouse vomeronasal organ (VNO) detects pheromones, which provide vital information needed to find a mate, detect predators, or alert to the presence of an intruder. Within the VNO, pheromones are sensed by two families of G-protein Coupled Receptors (GPCRs) the type 1 and type 2 vomeronasal receptors (V1Rs and V2Rs). However, little is known about the regulatory mechanisms that control the expression of V1R and V2R by vomeronasal sensory neurons (VSNs). Here we show that VSN precursors transiently express *Testes-expressed gene 15* (*Tex15*), which in the germline is required to repress the expression of transposable elements during spermatogenesis. We find that the absence of *Tex15* results in a dysregulated VR choice for V1Rs and A, B, and D family V2Rs, which manifest as a less diverse repertoire of VR expressing cells and includes a dramatic reduction in the expression of specific receptors that have been tied to intermale aggression. Accordingly, *Tex15* knockout mice exhibit lowered activation in the Accessory Olfactory Bulb (AOB) after exposure to male odorants, and a loss of stereotyped aggression between male mice. Taken together, these results show that *Tex15* plays a critical role in pheromone sensing by ensuring that VSNs express a diverse set of receptor proteins.

## Introduction

Olfaction plays a critical role in the survival and reproductive success of many terrestrial animals. The vomeronasal organ (VNO) is the key organ for detecting conspecific and allospecific pheromones from the environment, which can convey vital information such as another animal’s social position in the hierarchy, the receptivity of a female to mating, and the presence of unfamiliar intruder animals (Fu et al., 2015; Ibarra-Soria et al., 2014; Itakura et al., 2022; Leypold et al., 2002; Wysocki, 1979). Within the VNO, vomeronasal sensory neurons (VSNs) are responsible for detecting chemical signals and transmitting this information to the anterior olfactory bulb (AOB), from which it is relayed further on to the relevant neuronal circuits (Itakura et al., 2022). Mice with mutations that affect VSN function exhibit impaired social behaviour such as a lack of aggression in males or abnormal sexual behaviour in females (Ibarra-Soria et al., 2014; Itakura et al., 2022; Kondo et al., 2024; Leypold et al., 2002).

In mice, VSNs recognize chemical signals using G-protein coupled receptor proteins from the type 1 and type 2 families of vomeronasal receptors, known as the V1Rs and the V2Rs. The V1R genes are expressed in a monogenic and monoallelic fashion by VSNs in the apical layer of the epithelium, such that each apical VSN expresses only one type of receptor out of the approximately 230 genes in the V1R family (Dietschi et al., 2022; Hills et al., 2024). In contrast, the approximately 160 V2R genes get coexpressed in specific combinations by VSNs in the basal layer. The V2R genes are split between four sub-families: A, B, C, and D. Each type 2 VSN expresses one A, B, or D family gene, which encompasses approximately 160 genes total. Each VSN also expresses one or more genes from the C subfamily. The C subfamily includes seven members: *Vmn2r1–7*. Typically, a VSN will express either *Vmn2r1* or one or more of the other C subfamily members. There is a relationship between choice of an A, B, D gene and a C family expression such that individual A, B, D genes tend to be expressed together with either *Vmn2r1* or the other C family members. Expression of A, B, and D genes precedes expression of C genes, so the choice of an A, B, or D gene may instruct subsequent C family expression (Akiyoshi et al., 2018; Ishii & Mombaerts, 2011). In addition, in rodents a small number of VSNs express a receptor from a family of immune GPCRs, the formyl peptide receptors (Fprs). This family expanded in the rodent lineage after an ancestral Fpr was translocated into a VR gene cluster and gained expression in VSNs (Boillat et al., 2021; Dietschi et al., 2022).

The choice of a Type 1 or type 2 VR gene is specified over VSN differentiation. VSNs begin development at the marginal zones of the VNO as globose basal cells (GBCs) before becoming immature VSNs (iVSNs), at which point a differentiation between the apical (V1R-expressing) and basal (V2R-expressing) lineages occurs (Dietz et al., 2025; Hills et al., 2024). The mechanism behind this divergence involves multiple regulatory proteins, with differentiating basal cells expressing combinations of ATF5, Bcl11b, and Notch signalling factors. Perturbing these regulatory proteins can result in a disorganised VNO with a different distribution of apical and basal VSNs and VR choices (Katreddi et al., 2022; Lin et al., 2018; Nakano et al., 2015). However, beyond the specification of neuronal type, the mechanism that controls the choice of VR genes for expression remains unknown.

Here we show that *testis expressed gene 15 (Tex15)* is required for wild-type patterns of VR gene expression, VNO function, and stereotyped intermale aggression. We show that differentiating VSN progenitors transiently express *Tex15* RNA and protein. The peak of *Tex15* transcription coincides with the split between apical and basal VSNs lineages. Mice that are homozygous for a *Tex15* null allele exhibit altered expression of VR genes and altered abundances of neurons expressing individual VR genes, including VR genes associated with detecting male odors. When exposed to soiled male nesting material, *Tex15* null mice exhibit reduced neuronal activity in downstream neurons in the accessory olfactory bulb. Finally, male *Tex15* null mice fail to attack intruder males in a resident-intruder paradigm, and instead exhibit increased exploratory anogenital sniffing behaviour.

## Results

Single-cell RNA-seq has established *Tex15* as a marker of the immediate neuronal progenitor stage in another part of the olfactory system, the olfactory sensory neuron lineage in the main olfactory epithelium (Brann et al., 2025; Pourmorady et al., 2024), but its expression in the VSN lineage has not been characterized. To determine whether *Tex15* is expressed at any point during VSN differentiation, we examined previously published single-cell RNAseq data from mouse VNO (Hills et al., 2024). This dataset contains eight mice, four at postnatal day 56 (P56) and four at postnatal day 14 (P14). Since we were interested in examining the immature VSNs, we used the P14 samples, which have a higher count of immature VSNs but otherwise resemble the P56 samples. This cohort has two males and two females. Consistent with prior work, UMAP analysis generates distinct clusters of cells expressing markers of mature apical and basal VSNs as well as clusters corresponding to various stages of VSN progenitors (Figure S1A). We excluded a cluster of atypical cells, noted by Hills et al, that express markers of both apical and basal VSNs, since these cells do not express *Tex15* and their connection to the canonical set of VSN progenitors is uncertain.

**Figure 1.**
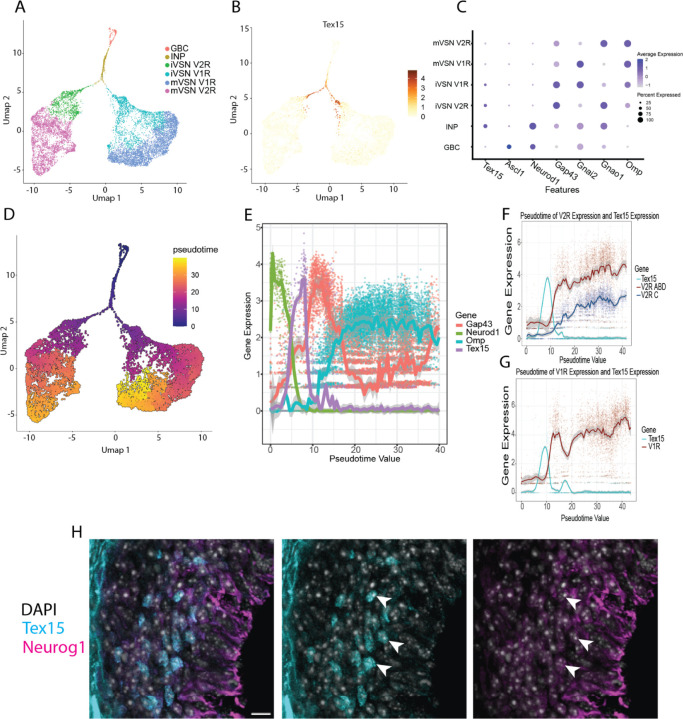
*Tex15* is transiently expressed by VSN progenitors before VR choice. (A) Labelled UMAP of the neuronal VSN lineage. (B) *Tex15* expression is highest during the differentiation of apical and basal VSNs. (C) *Tex15* is exclusively expressed during the INP and iVSN stage. (D) Pseudotime analysis UMAP of the neuronal lineage (E) Plot of neuronal development markers with *Tex15* showing *Tex15* expression peaks between *Neurod1* and *Gap43*. (F) *Tex15* expression peaks just before V2R expression rises (G) *Tex15* expression peaks just before V1R expression rises. (H) *Tex15* protein is present in postnatal day two (P2) pups in the nuclei of cells in the marginal zones scalebar:10μm.

*Tex15* expression is restricted to cells in the neuronal lineage (Figure S1B C). Thus, to examine *Tex15* expression over VSN differentiation, we isolated cell clusters corresponding to the neuronal lineage markers and reclustered these cells. We then assigned each cluster to a stage of differentiation based upon the expression of known marker genes, namely: *Ascl1* for early progenitors, *Neurod1* for immediate neuronal progenitors, *Gap43* for immature VSNs, *Gnai2* for mature apical VSNs, and *Gnao1* for mature basal VSNs (Figure 1A). Notably, *Tex15* expression peaks in the INP population coinciding with the divergence of INP cells into the apical and basal differentiation lineages (Figure 1B,C).

To examine the precise timing of *Tex15* expression, we conducted pseudotime analysis of the neuronal lineage (Figure1D). Consistent with the cell stage analysis, *Tex15* expression peaks after the peak of expression of Neurod1 and prior to the peak of expression of Gap43 (Figure 1E). Thus, we asked whether *Tex15* expression similarly aligns with the onset VR gene expression in the VSN lineage. To accomplish this, we plotted *Tex15* and VR expression for each lineage separately against the pseudotime values generated for the cells. In the basal VSN lineage, we took the additional step of dividing VR genes based upon assignment to the C subfamily or the A, B, and D subfamilies, which exhibit different patterns of expression in mature cells (Figure 1F). We observed that *Tex15* expression precedes A, B, D, and C subfamily expression. Notably, we also observed a clear temporal difference between the expression of A, B, and D subfamily V2Rs and C subfamily V2Rs. In the apical lineage, *Tex15* expression peaks prior to the expression of V1R genes (Figure 1G). Expression of the A, B, and D V2Rs rises shortly after *Tex15* expression, roughly aligning with the onset of Gap43. In contrast, expression of the C subfamily only begins to rise towards the latter half of the pseudotime line. These observations are consistent with a prior study that observed that expression of A, B and D subfamilies of V2R genes was detectable by RNA in situ hybridisation (ISH) in immature cells prior to the expression of a C subfamily gene (Ishii & Mombaerts, 2011). We can conclude that *Tex15* expression is tightly restricted to a time point in the development of VSNs, which precedes VR expression.

We next sought to determine whether *Tex15* protein is present in immature VSNs in the VNO. To accomplish this, we analyzed *Tex15* expression in postnatal day 8 mice bearing a Neurog1-GFP reporter gene that labels INPs in the marginal zones. We performed a fluorescent immunohistochemistry assay using a mouse monoclonal antibody for *Tex15* and a GFP antibody to enhance the endogenous GFP signal. Aside from non-specific staining along the basal membrane, *Tex15* immunoreactivity is restricted to the nuclei of cells in the marginal zones of the VNO, all of which also exhibit labeling with Neurog1-GFP (Figure 1H). In summary, immunohistochemistry confirms the presence of TEX15 protein at the timepoint just preceding VR expression in immature cells.

**Figure 2.**
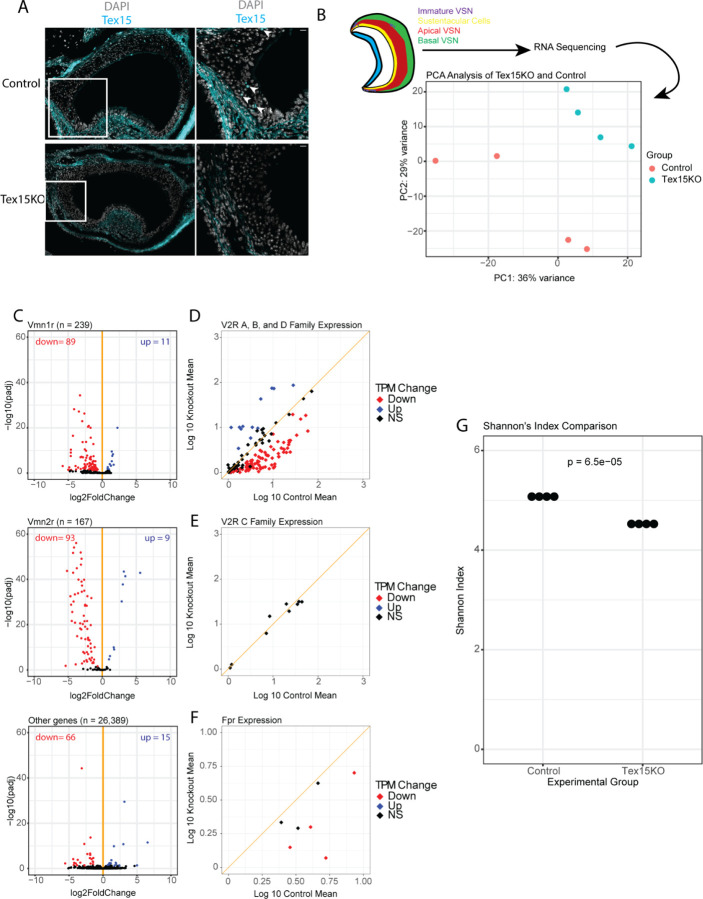
*Tex15* knockout mice have altered VR expression and reduced diversity. (A) Coronal sections of the VNO of postnatal day 2 P2 littermates confirm that *Tex15* is knocked out in the transgenic line. scalebar: 10μm. (B) PCA analysis of whole VNO Bulk-RNAseq libraries. (C) Loss of *Tex15* disproportionately affects VR expression. Differentially expressed genes are red (down) or blue (up) (padj < 0.05 for log2FC > 1.5) wald test. (D) The A, B, and D family of V2R receptors are significantly impacted by the loss of *Tex15*. (E) The C family of V2Rs is not impacted by the loss of *Tex15*. (F) Fpr receptor diversity is greatly lowered by the loss of *Tex15*. (G) Shannon’s Index of the Control and Tex15KO libraries. T test

To examine the effects of *Tex15* we used a *Tex15* null allele (Tex15KO) in which the majority of the *Tex15* coding sequence has been replaced by a beta galactosidase cassette (Yang et al., 2008). Homozygous Tex15KO mice and littermate controls were obtained by crossing Tex15 +/− heterozygous mice. To confirm that the knockout lacks *Tex15* expression in the VNO, we performed a *Tex15* immunofluorescence staining in postnatal day 2 KO mice and littermate controls (Figure 2A). As in adult mice, *Tex15* immunoreactivity is present in the marginal regions of the VNO, but we also observe sparse immunoreactivity throughout the rest of the VNO in heterozygous and wildtype animals. This signal is completely absent in the knockout. Otherwise, the overall structure and organization of the VNO appear intact in these animals.

To determine whether the absence of *Tex15* impacts VR gene expression in the VNO, we performed RNAseq on VNO tissue obtained from adult Tex15KO mice and heterozygous controls. We sequenced poly-A selected RNAseq libraries from four animals per genotype, and then performed differential gene expression analysis using DESeq2 (Love et al., 2014). As expected, the samples cluster by genotype when subject to principal component analysis (Figure 2C). Differential expression analysis identifies 283 genes that exhibit a greater than 50% change in expression between KO and Het animals (padj < 0.05 and log2 fold change > 1.5-fold). Remarkably, the majority of differentially expressed genes are V1Rs and V2Rs: 151 out of 283 genes. Notably, overall expression of V1R versus V2R genes is not affected; the total sum of transcript counts from each family are similar between Het and KO animals (Figure S2A). Rather, the expression of individual receptors is significantly different, with some exhibiting increased expression and many more exhibiting decreased expression. In particular, within the V2R family the A, B, and D subfamilies, 102 out of 160 genes are differentially expressed in basal VSNs, whereas none of the seven co-expressed genes from the C family have a significant differences between the control and experimental group (Figure 2D, E). Interestingly, we also see reduced expression of more than half of the FPR genes, with 4 significantly decreased in Tex15KO mice (Figure 2F).

Since most V1R and V2R genes exhibit reduced expression in the VNO of Tex15KO animals, this should result in a reduction in the diversity of the VR gene repertoire. We quantified VR gene diversity by calculating the Shannon diversity index value for VR gene expression levels in Tex15KO and control samples. We observe a significant reduction in these scores, indicating reduced VR diversity in KO mice (Figure 2G).

VR genes are arranged in clusters across chromosomes, and this clustered organization contributes to their regulation (Dietschi et al., 2022). We asked whether the altered expression of VR genes in Tex15KO animals reflected the chromosome cluster location of each VR gene. Grouping VR genes by cluster did not reveal any discernable patterns to which VRs were upregulated and which were downregulated. Individual VR clusters frequently contain both upregulated and downregulated VR genes, nor are there individual VR clusters that dramatically (Figure S2B C). The most notable non-VR genes that were impacted were the H2-MV group of genes, which have previously been reported to be expressed in specific combinations with VR genes. Four H2-MV genes exhibited significantly reduced expression in Tex15KO VNOs: *H2-M1*, *H2-M9*, *H2-M11*, and *H2-M10.4* (Figure S2D). The first three are notable, as they are known to tightly coexpress with *Vmn2r82* and *Vmn2r81* (Devakinandan et al., 2024) which are downregulated in the knockout (Figure S2E). This suggests that their expression could be dependent on the expression of their associated A, B, and D family V2R.

**Figure 3.**
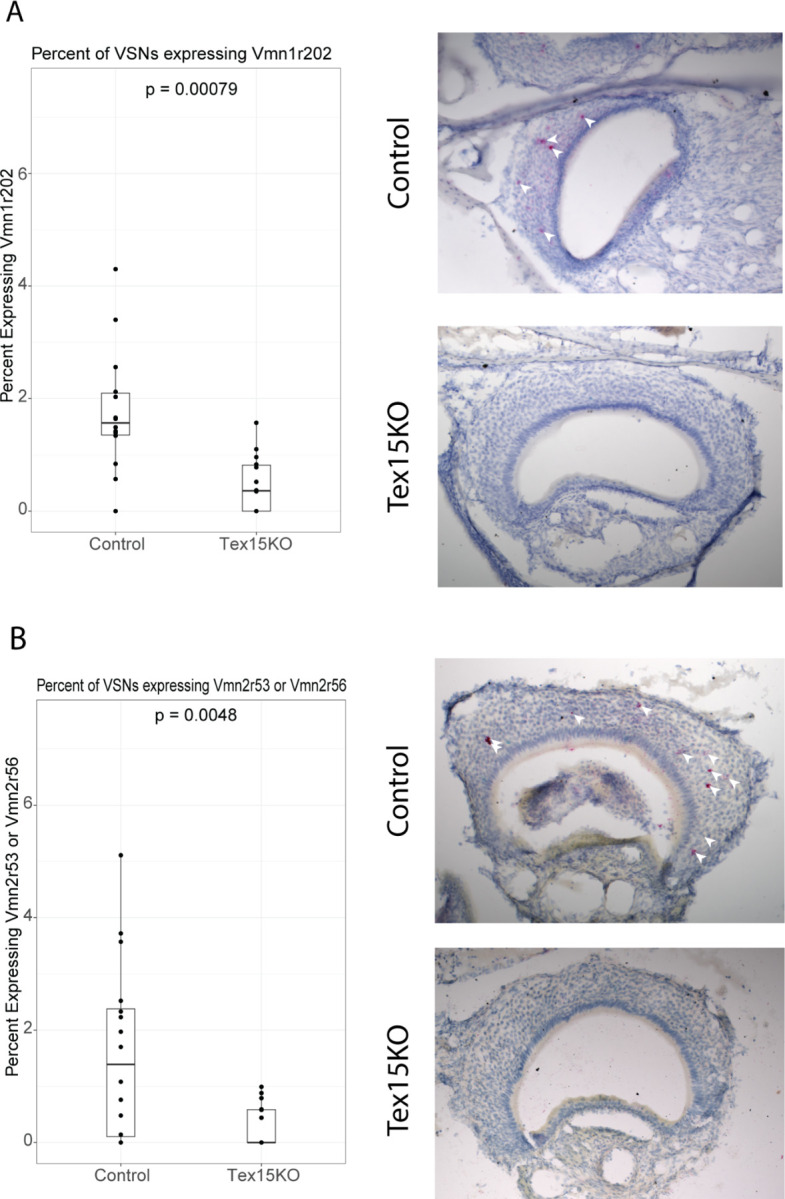
The lower transcription of VRs results in VSNs choosing those VRs at lower frequencies. (A) The percent of VSNs expressing Vmn1r202 in knockout vs control sections (p= 0.00079) with accompanying images of a knockout and control example. Signal in red. *(B)* The percent of cells within the VSN layer expressing either *Vmn2r53* or *Vmn2r56* in a knockout vs control (p= 0.0048) with accompanying images of a knockout and control example signal in red. For bulk RNA-seq, n=4 per genotype, and p value is calculated by wald test). For percent of cells expressing VR genes, n=3 per genotype per probe, p values calculated with t test

Since V1R and V2R A, B, and D genes are expressed monogenically within the population of VSN, the changes we observed by bulk RNAseq could arise from either a change in the number of cells expressing each VR genes, reflecting a change in the frequency of VR choice, or a change in the levels of VR expression within the same number of cells. To test whether differentially expressed VR genes are expressed by a different number of cells in Tex15KO animals, we performed RNA in situ hybridization (ISH) to label individual cells expressing specific VR genes in tissue sections from the VNO. Due to the very high sequence similarity among VR genes, we relied on a chromogenic ISH strategy (BaseScope) that can target very short sequences, as few as 50 bases. For these analyses we focused on a pair of VR genes with reduced expression in Tex15KO, *Vmn1r202* (log2fold change = −3.45, padj = 4.89e-35) and *Vmn2r53* (log2fold change = −1.25, padj = 8.29e-4) with p values generated with a wald test. Due to the high degree of similarity between VR genes, the probe for *Vmn2r53* also targets *Vmn2r56* which is also transcriptionally downregulated (log2fold change = −3.93, padj = 1.42e-21). As a positive control for the BaseScope assay, we also examined the expression of a C family VR, *Vmn2r5*, that is not differentially expressed (log2fold change = 0.09, padj = 0.20). We dissected the VNOs of Tex15KO and littermate controls and counted the nuclei present in the epithelial layer to see what percentage of nuclei were labeled by each probe. As expected the *Vmn2r5* probe is widely expressed in the basal layer of the VNO (Figure S3 A), and is similar in Tex15KO and control tissue sections. In contrast, the frequency of nuclei expressing the *Vmn2r53* and *Vmn1r202* was significantly different by t test in Tex15KO mice, in accordance with the transcriptional changes observed by bulk RNA-seq of Tex15KO and control VNO (Figure 3A, B). Importantly, we still observe strongly positive cells in the KO animals (Figure S3 B), but at a reduced rate compared to control tissue, which is consistent with an alteration in receptor choice rather than expression per cell. This confirms that the transcriptional difference seen in the bulk RNAseq translates into a lowered frequency of that receptor being expressed by neurons.

**Figure 4.**
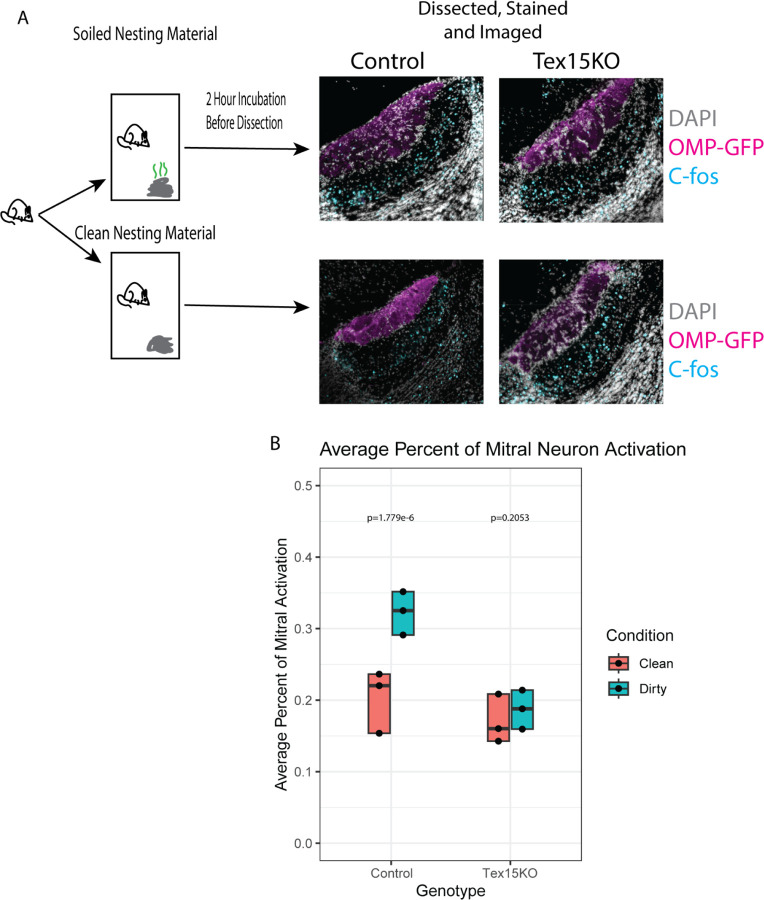
Loss of *Tex15* and VR diversity results in lower activation in the AOB. (A) The c-fos assay diagram and images. (B) Comparing the average percent of activated neurons between the various experimental groups with the wild type in dirty having the highest activation (32.3%) and the knockout in clean having the lowest activation (17%). P values were calculated by t test.

Our prior results indicate that Tex15KO VNOs contain a reduced diversity of VSNs. Since the presence of a diverse repertoire of VR genes present in the mouse genome is thought to facilitate the detection of signals from conspecifics, we asked whether the reduced VSN diversity in Tex15KO mice might impair the response to mouse derived odorants. We tested this hypothesis by quantifying the expression of cfos, an immediate early gene that is expressed in response to neuronal activity in the mitral cells of the accessory olfactory bulb. These are the first layer of cells downstream of VSN and serve to process the information encoded in pheromones and relay it to the downstream targets in the brain (Imamura et al., 2020). To test this, *Tex15* knockout mice and wildtype controls were placed in a new cage that contained either soiled male nesting material or clean nesting material for 2 hours, then were assayed for c-fos expression. (Figure 4A). To facilitate identification of the AOB, these experiments were performed with mice bearing an OMP-Ires-GFP reporter gene, which labels VSN axons located within the glomeruli of the Accessory Olfactory Bulb (AOB). As expected, wild type mice exposed to dirty bedding had significantly higher rates (p = 1.779e-06, t test) of c-fos positive nuclei (32.3%± 2.63%) than wildtype mice exposed to clean bedding (20.3% ± 3.79%). In contrast, *Tex15* knockout mice exposed to dirty bedding have similar rates (p = 0.205, t test) of cfos activation (18.7% ± 2.36%) to knockout mice exposed to clean bedding (17% ± 2.95%), and both of these conditions resemble wild type mice exposed to clean bedding. Thus *Tex15* knockout mice in a stimulus-rich environment exhibit a similar level of activation to control mice in a clean environment. These results suggest that the alterations in VR expression rates observed in Tex15KO mice impair the response of the vomeronasal system to male derived odorants.

The impaired activation of the AOB in response to male odorants suggests that Tex15KO mice may also exhibit altered behavior in response to male cues. The VNO plays a critical role in many innate behaviours, such as intermale aggression or identifying the sex of the other mouse (Leypold et al., 2002). *Vmn2r53*, which has dramatically reduced expression in Tex15KO mice (Fig 3B), has previously been implicated in aggressive behavior (Itakura et al., 2022). To test VNO function in Tex15KO mice we chose the resident intruder experiment, as a functioning VNO is key to eliciting the stereotyped aggression from the male resident (Itakura et al., 2022; Leypold et al., 2002). A male mouse in an established territory will react aggressively toward an unknown male intruder (Leypold et al., 2002). This assay typically uses sexually experienced males as the resident, as they exhibit greater responses than sexually naive males (Koolhaas et al., 2013). However, due to widespread changes in VR receptor diversity, we chose to use sexually naive males for these experiments, to avoid potentially confounding effects of *Tex15* on mating behavior.

**Figure 5.**
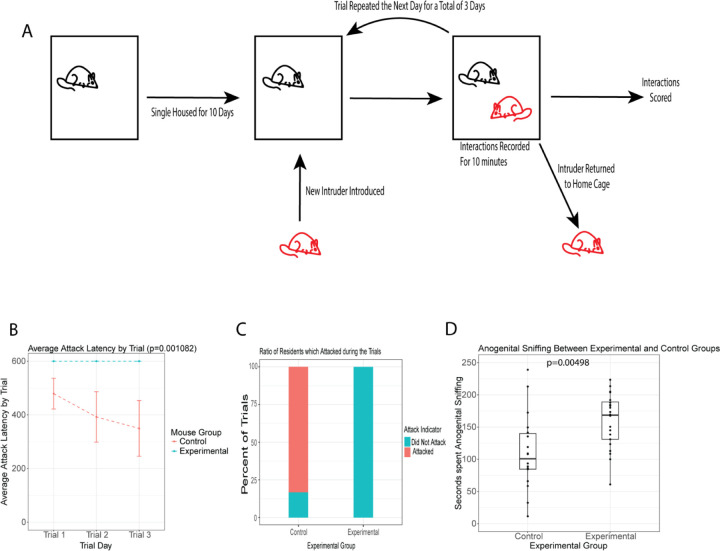
*Tex15* knockout males do not show aggressive behaviour towards intruders. (A) Diagram of the resident intruder assay conducted on the mice. (B) Average attack latency of each experimental group across all 3 trials with standard mean error. If the resident did not attack, the attack latency was set at 600 seconds. (C) Percent of trials in which the resident attacked the intruder. (D) Plot of the average time spent by the resident anogenitally sniffing the intruder.

We ran the resident intruder with three 10-minute trials over the course of three days while comparing control and *Tex15* knockout naive males interacting with intact intruders that are age-matched (Figure 5A). An attack latency of 600 seconds indicates that the resident did not initiate an attack during the trial. Control residents showed the typical pattern of behavior, with a shortening attack latency over the course of the three trials (Itakura et al. 2022). However, Tex15KO males exhibited very different behavior; Tex15KO male residents never initiated an attack against an intruder at any point during any of the trials (Figure 5B, C). We noted that the knockout residents spent significantly more time anogenitally sniffing their intruders when compared to the control (Figure 5D). This raises the possibility that Tex15KO residents may not receive sufficient stimulus to trigger the stereotyped aggression behaviour and thus spends more time during the trial trying to acquire enough stimulus to trigger an appropriate response.

## Discussion

We find that *Tex15* is expressed at a critical time point in VSN development, coinciding with the divergence between the basal and apical populations and shortly preceding the onset of VR gene transcription. In mice lacking *Tex15*, the overall expression of VR genes is unchanged, but the relative abundance of individual VR genes is altered. Most VR genes exhibit reduced expression, whereas a few are expressed at higher levels, resulting in a reduction in the overall diversity of VR gene expression. For two of the downregulated VR genes, we show that reduced expression corresponds to a reduced number of cells expressing these VR genes. These transcriptional changes are accompanied by reduced AOB activation in response to soiled male nesting material. We also observe that Tex15KO mice exhibit a lack of stereotyped intermale aggression and increased ano-genital sniffing behaviour. Taken together, these findings indicate that *Tex15* has a crucial role in ensuring that VR receptor choice results in diverse outcomes, generating a VNO in which many subtypes of VSN are represented.

The transient expression of *Tex15* during a precise developmental window, coupled with the striking alterations in VR gene expression observed in *Tex15* knockout mice, implicates Tex15 as a key regulator of VR gene choice. The loss of *Tex15* affects V1Rs as well as V2R families A, B, and D, implying these receptors share a *Tex15*-dependent choice mechanism. Conversely, the V2R C family remains unaffected, suggesting they utilize a distinct regulatory pathway.

What then is the role of *Tex15* in regulating VR gene choice? In testes, *Tex15* associates with PIWI family proteins MILI and MIWI2, which mediate piRNA-directed methylation and silencing of transposable elements. Tex15KO mice exhibit reduced DNA methylation of LINE1 and LTR family transposable elements in testes, which is accompanied by increased transposon expression (Schöpp et al., 2020; Wang et al., 2005; Yang et al., 2020). Expression of PIWI family members is very low or absent in the VNO, making it unlikely that the altered VR expression results from altered piRNA directed gene regulation. However, it is possible that *Tex15* associates with the gene silencing machinery that carries out the DNA methylation and heterochromatin deposition on transposable elements that are targeted by piRNAs. This would implicate a role for heterochromatin deposition in regulating VR gene choice, similar to what has been proposed for the olfactory receptor genes (Bashkirova et al., 2023; Magklara et al., 2011). These results also suggest a possible role for small RNA directed silencing in VR gene regulation, which has an incompletely understood role in the olfactory system (D. Yang et al., 2020).

Alternatively, it is possible that the observed effects on VR gene expression are a direct result of dysregulation of transposable elements in Tex15KO mice. The transcription start sites (TSSs) of over half of VR genes were found to be upstream of LINE1 elements and, moreover, these LINE1 elements are transcribed (Pascarella et al., 2014). It may be that this LINE1 transcription influences subsequent VR gene choice, and that alterations in this transcription in Tex15KO mice results in the observed changes in VR gene expression. In this scenario, variation in the proximity of transposable elements to the transcriptional start site of VR genes accounts for the variable effect of the Tex15KO on VR gene expression.

We observe striking defects in the function of the vomeronasal system in Tex15KO mice. Indeed the responses to male bedding observed in the accessory olfactory bulb of Tex15KO mice are comparable to those from wildtype mice housed in a clean environment. It is unclear whether these differences arise directly from the altered repertoire of expressed VR genes, or some other defect in VSN signaling in Tex15KO mice. However, this loss of sensitivity to male odorants aligns with a dramatic reduction in aggressive behavior in Tex15KO male mice in a resident-intruder behavioural assay. Tex15KO male mice never attacked a male intruder across any of our trials. This loss of aggressive behaviour was accompanied by a significant increase in anno-genital sniffing behaviour. We speculate that this increased exploratory behaviour may reflect a response to deficient signaling through the VNO pathway.

We show that *Tex15* expression is restricted to a key stage of VSN differentiation, corresponding to the split between the V1R and V2R lineages, and directly preceding the onset-of VR gene expression. As such, *Tex15* could serve as an excellent marker for this crucial stage of differentiation. A large portion of the literature has focused on this time period in VSN development (Devakinandan et al., 2024; Enomoto et al., 2011; Katreddi et al., 2022; Lin et al., 2018). A *Tex15* Cre transgene could be developed and used to knockout specific elements at the exact timepoint of differentiation or activate a fluorescent reporter. Uncovering *Tex15* as both a marker for VSN differentiation for future studies delving into the subject and as a key component to the VR choice mechanism in the VSN are important steps in advancing our knowledge of the VNO. Future direction for the research would be determining what protein partners associate with *Tex15* to determine its specific mechanistic function in the VNO.

## Methods and Materials

### Tex15 Immunofluorescence

Mice under P8 were decapitated and their heads were fixed in 4% PFA in PBS for 30 minutes at room temperature. The heads were then washed 3 times for 5 minutes in PBS before being submerged in a 30% sucrose and PBS mixture at 4C for 3 days. Afterwards the heads were embedded coronally in Optimal Cutting Temperature (cat#:25608–930) and sectioned with a cryostat to a thickness of 14 microns and the sections put on a slide. The slides were dunked in a solution of 0.1% PBSTriton before applying a primary antibody solution of a monoclonal Tex15 antibody (1H1A324–3) diluted at 1:10 in 4% goat serum and 0.1% PBST. The slide was incubated in a humidified chamber at 4C overnight. On the second day the slides were washed 3 times for 5 minutes with 0.1% PBST. The secondary antibody Goat anti mouse IgG1 Cy3 (catalogue#: 115-165-205) and DAPI (Sigma-Aldrich: MBD0015–1ML) were diluted at 1:1000 in 4% goat serum 0.1% PBST and applied on the slide. The slide was incubated in a humidified chamber at room temperature for 1 hour. The slides underwent three more five minute washes in 0.1% PBST. The slides were mounted with VectaShield Hardset Mounting Medium and left to cure for 15 minutes in the dark. The images were then taken with a Leica TCS SP8 tauSted 3X confocal and tilescanned at 40x. Tilescan images were then stitched together in FIJI with the Grid/Collection Stitch plugin (Preibisch, et al, 2009).

### Tex15 and Ngn-GFP Immunofluorescence

Mice under P8 were decapitated and their heads were fixed in 4% PFA in PBS for 30 minutes at room temperature. The heads were then washed 3 times for 5 minutes in PBS before being submerged in a 30% sucrose and PBS mixture at 4C for 3 days. Afterwards the heads were embedded in Optimal Cutting Temperature (cat#:25608–930) and sectioned with a cryostat to a thickness of 14 microns and the sections put on a slide. The slides were blocked for 1 hour in a solution of 4% Goat Serum(Cat#: G9023) 0.1% PBSTriton before applying a primary antibody solution. The primary antibodies used are a monoclonal Tex15 antibody (1H1A324–3) diluted at 1:10 and anti-GFP(Cat#: gfp-1020) at 1:1000 to enhance the endogenous fluorescence in 4% goat serum and 0.1% PBST. The slide was incubated in a humidified chamber at 4C overnight. On the second day the slides were washed 3 times for 5 minutes with 0.1% PBST. The secondary antibody Goat anti mouse IgG1 Cy3 (catalogue#: 115-165-205), IgY Goat anti chicken Alexa Fluor 488 (Cat#: A11039) and DAPI (Sigma-Aldrich: MBD0015–1ML) were diluted at 1:1000 in 4% goat serum 0.1% PBST and applied on the slide. The slide was incubated in a humidified chamber at room temperature for 1 hour. The slides underwent three more five minute washes in 0.1% PBST. The slides were mounted with VectaShield Hardset Mounting Medium and left to cure for 15 minutes in the dark. The images were then taken with a Leica TCS SP8 tauSted 3X confocal at 63x.

### C-fos Imaging and Counting

Mice were placed in a clean cage for 10 minutes to habituate. After the 10 minutes soiled nesting material from male cages or clean nesting material was placed into the cage with them and the mice were incubated for 2 hours with the material. The mice were euthanised with CO2 and their olfactory bulbs were dissected out and fixed on ice for 20 minutes with 4% PFA in PBS. The bulbs would undergo 2 PBS washes on ice before being placed in 50% 0.5M EDTA in PBS at 4C overnight then in a solution of 30% sucrose in PBS at 4C for 3 days or until the tissue sank to the bottom. The bulbs were embedded sagittally in OCT and sectioned on a cryostat at 14 microns.

The slides were blocked in a solution of 4% donkey serum and 4% goat serum in 0.1% PBSTriton for one hour. They were incubated in the primary antibody solution of c-fos (Cat#: ab190289) at 1:500 and anti GFP (Cat#: gfp-1020) at 1:1000 to enhance the fluorescence overnight in a humidified chamber at 4C. On the second day the slides were washed 3 times for 5 minutes with 0.1% PBST. The secondary antibody donkey anti rabbit alexa 555, Alexa Fluor 488 (cat#:A21206) and DAPI (Sigma-Aldrich: MBD0015–1ML) were diluted at 1:1000 in 4% goat and donkey serum in 0.1% PBST and applied on the slide. The slide was incubated in a humidified chamber at room temperature for 1 hour. The slides underwent three more five minute washes in 0.1% PBST. The slides were mounted with VectaShield Hardset Mounting Medium and left to cure for 15 minutes in the dark.

Three knockouts and three wildtypes were put through each condition for a total of twelve mice. Three spaced 14-micron-thick sections of their AOB were then imaged on the Widefield Leica for a total of 36 images for the dataset. The photos were tilescans taken at 40X with a Widefield Leica DMI8 Thunder System. We imaged the glomerular and mitral layer of the AOB and counted the number of c-fos positive nuclei relative in the mitral layer and compared them to the total nuclei count of the mitral layer. Nuclei in the mitral layer were considered positive for c-fos expression if all the euchromatin of a nucleus was occluded by c-fos expression on the same focal plane as the nucleus. FIJI counting plugin was used for counting cfos expressing nuclei in the mitral layer of the accessory olfactory bulb. R programming was used for data analysis. A t test was conducted on the average percent positive nuclei between the two groups to calculate significance.

### Basescope Imaging and Counting

The basescope probes were generated by ACD and the protocol used was the fixed/frozen protocol as written for the basescope duplex and basescope single channel assay. The Basescope Images were taken on a Zeiss Axio Imager.A1 with a lumineer camera with RGB channels for colour brightfield imaging at 20X. The basescope images were analyzed to determine the number of nuclei that were red positive relative to negative nuclei. The specifications for a positive versus a negative cell were determined by comparing experimental basescope images to images that used a negative control bacterial probe provided by ACD. The signal in the negative images served as a standard for background signal. A positive nucleus required signal strength higher than the negative control in a concentrated area surrounding or adjacent to a nucleus. FIJI counting plugin was used for counting expressing nuclei in the epithelial layer of the VNO. R programming was used for data analysis. A t test was conducted on the percentage positive nuclei in the sections between the two groups.

### RNA Extraction and sequencing

The VNO would be dissected and placed in 1 ml of Tryzol for 10 minutes or would be frozen at −80C and thawed later for the next step. The RNA was isolated using the Zymo Research Direct-zol RNA Miniprep Kits (Cat# R2050). The RNA isolate was nanodropped on a Thermo Fisher Scientific Nanodrop One for concentration. It was then quality controlled for RIN in an Agilent Tapestation 4150. Samples which had >7 RIN were sent to Admera (South Plainfield, NJ, USA) for DNAse treatment and poly A tail sequencing. The sequenced samples were analysed with the DEseq2 package in R programming and significance was found with a wald test.

### Resident Intruder

Resident males were isolated for a period of 10 days prior to the trials. The resident males are naive and have never been housed with a female. A wildtype, naive intruder of similar age and size to the resident would be placed into the resident’s cage and their interactions recorded for 10 minutes and scored for social interactions, anogenital sniffing, and aggression based on how many seconds they spent engaging in each behaviour. The trial is repeated 3 times over the course of 3 days, each time with a new intruder who has not been used in the protocol. The recordings were scored by two independent scorers and averaging the values and scores were generated by determining how many seconds the residents spent engaging in certain behaviours. The scores between knockout residents and wildtype residents were then compared. A t test was conducted on the average attack latency between the two groups and the time spent ano-genital sniffing between the two groups.

## Supplementary Material

Supplement 1
