# Supplementary material for: Tex15 is required for vomeronasal sensory neuron diversity and male pheromone detection
Source: bioRxiv. 2025 Dec 10:2025.12.03.690614. Preprint. [Version 2] doi: 10.64898/2025.12.03.690614 (PMC12710957; doi:10.64898/2025.12.03.690614)
Supplement: Supplement 1 [file media-1.pdf]

Figure S1

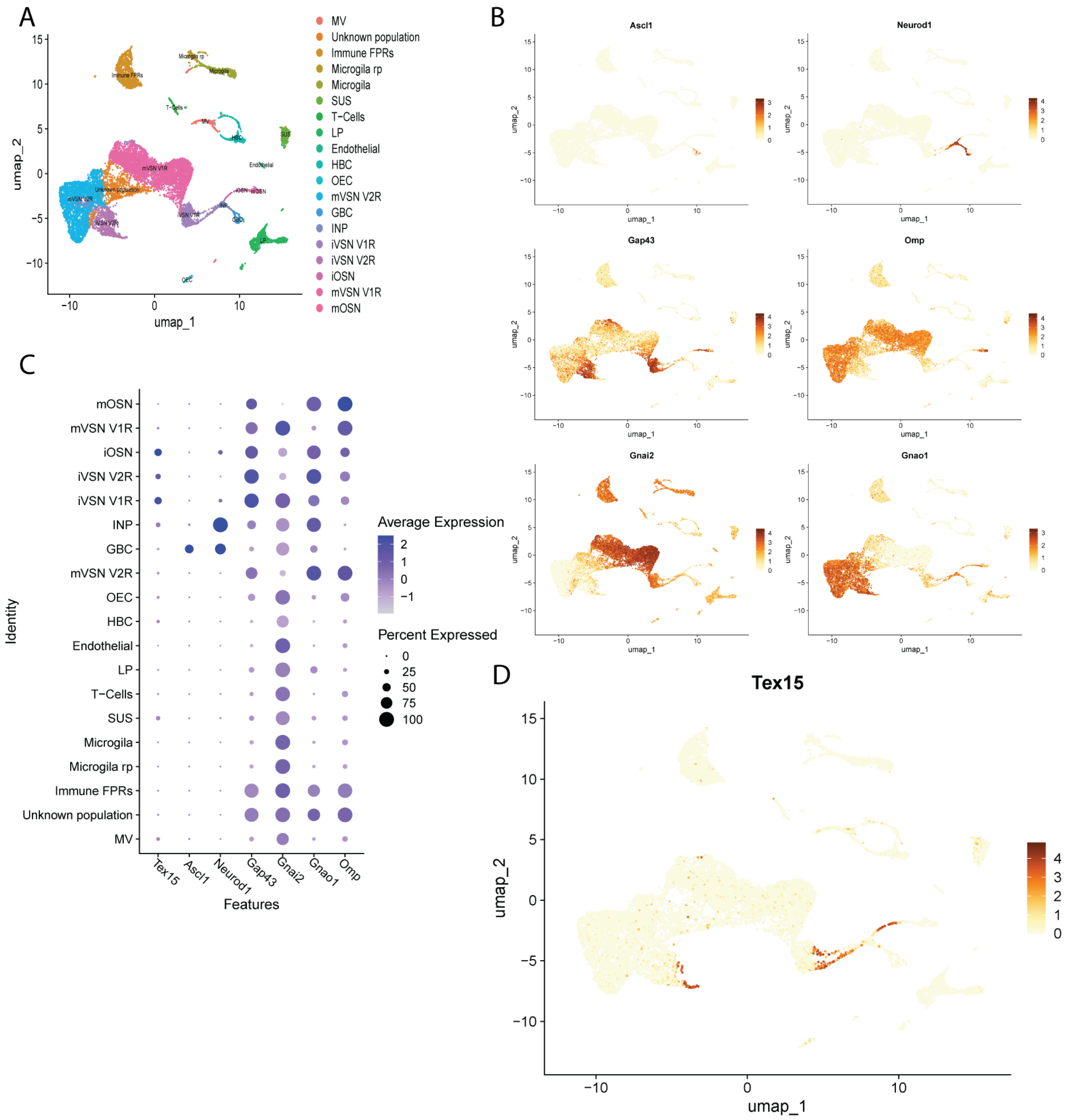

Figure S1 (A) UMAP of the clusters of the VNO cell populations. (B) markers for the neuronal lineage in the VNO. (C) Dot plot of marker and *Tex15* expression in the clusters demonstrating that *Tex15* is limited to the neuronal lineage. (D) UMAP of *Tex15* expression in the VNO

Figure S2

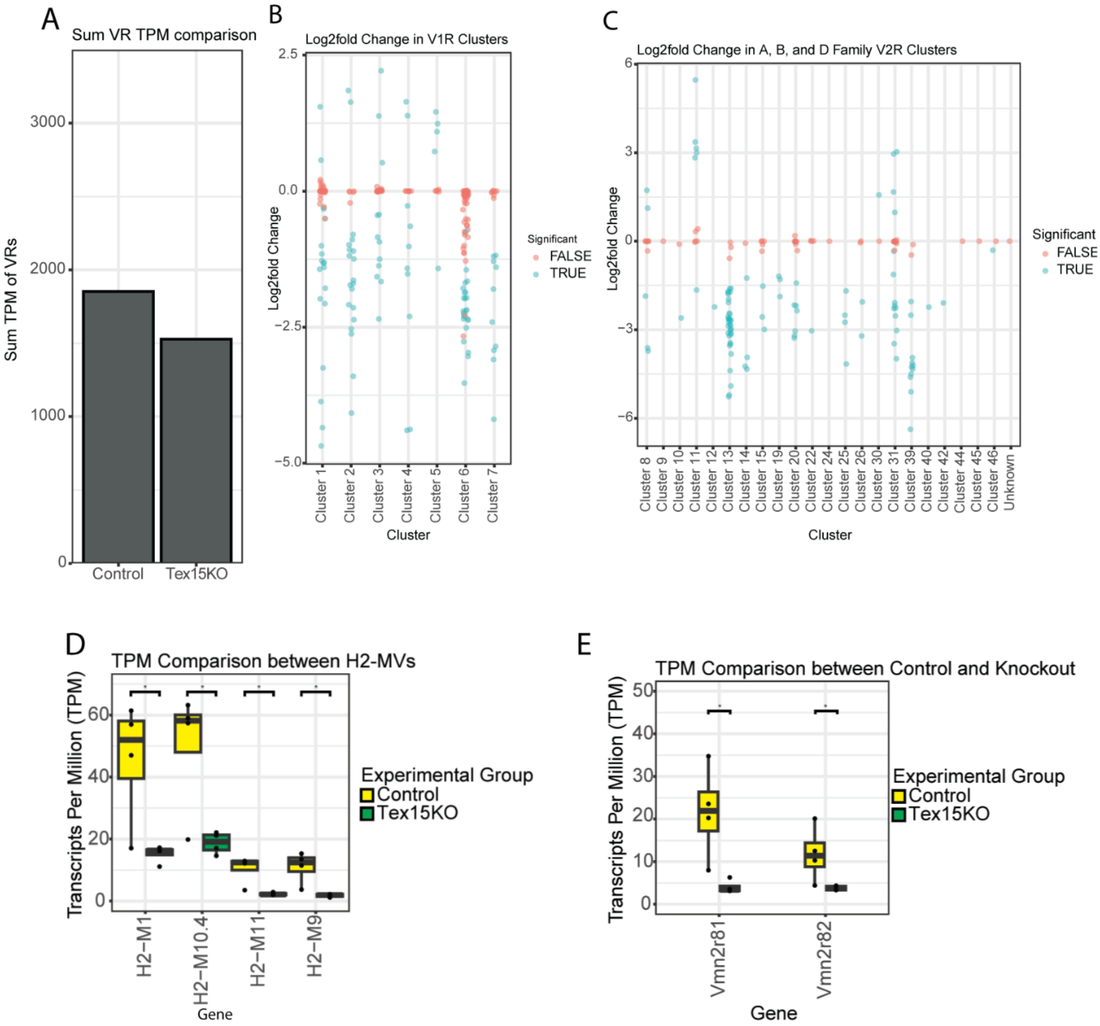

Figure S2 (A) Sum VR TPM comparison between the control and experimental group. (B) Log2fold change of V1Rs grouped by cluster blue= significantly affected, red=not significantly affected. (C) Log2fold change of A, B, and D family V2Rs grouped by cluster blue= significantly affected, red=not significantly affected.. (D) Certain H2-MV genes are affected by the loss of *Tex15* yellow= control, green= knockout. (E) *Vmn2r81* and *Vmn2r82*, which strongly coexpress with certain H2-MV genes, are downregulated in the knockout yellow= control, green= knockout.

Figure S3

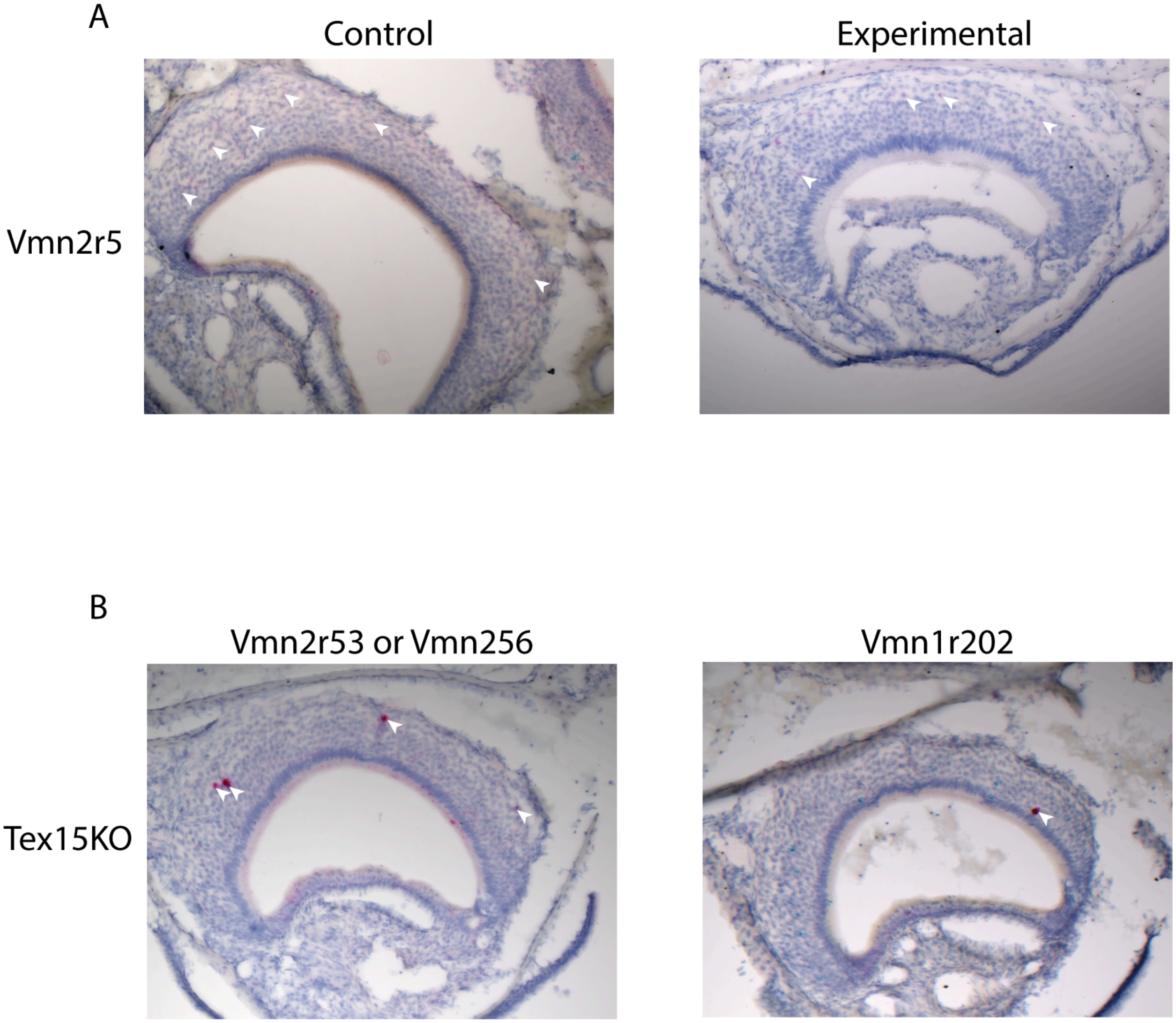

Figure S3 (A) Representative images of Vmn2r5 expression in the control and experimental mice (B)Representative images of downregulated VR expression in the Experimental group
